# Taxonomic revision of the endemic Bornean genera *Anexodus* Pascoe and *Pantilema* Aurivillius (Coleoptera, Cerambycidae, Lamiinae)

**DOI:** 10.3897/zookeys.669.12608

**Published:** 2017-04-20

**Authors:** Radim Gabriš, Filip Trnka, Rodzay Abdul Wahab, Robin Kundrata

**Affiliations:** 1 Department of Ecology and Environmental Sciences, Faculty of Science, Palacky University, Šlechtitelů 11, Olomouc, Czech Republic; 2 Universiti Brunei Darussalam, Institute for Biodiversity and Environmental Research, Jalan Tungku Link Gadong, BE1410, Brunei Darussalam; 3 Department of Zoology, Faculty of Science, Palacky University, 17. listopadu 50, 771 46, Olomouc, Czech Republic

**Keywords:** Brunei, diversity, endemism, hot-spots, longhorn beetles, Malaysia, Morimopsini

## Abstract

The genera *Anexodus* Pascoe, 1866 and *Pantilema* Aurivillius, 1911 (Cerambycidae: Lamiinae: Morimopsini), both endemic to Borneo, are revised. Four species of *Anexodus* are recognized: *A. aquilus* Pascoe, 1886 (Malaysia: Sabah), *A. sarawakensis* Sudre, 1997 (Malaysia: Sarawak), *A. syptakovae* **sp. n.** (Malaysia: Sarawak), and *A. tufi* **sp. n.** (Brunei). *Pantilema* is a monotypic genus containing *P. angustum* Aurivillius, 1911 (Malaysia: Sarawak) which is known only from the holotype. For the first time, genital structures are studied in these genera. An identification key for the species of *Anexodus* is provided and their intraspecific morphological variability and distributions are discussed.

## Introduction

Cerambycidae forms one of the largest and most well-known beetle lineages in the world (Švácha and Lawrence 2014, Nearns et al. 2017). However, some lineages, especially those from the tropical regions, are underinvestigated, with numerous new taxa described recently (e.g. Bezark et al. 2016, Bi and Lin 2016, Huang and Lin 2016, Ohbayashi et al. 2016, Santos-Silva et al. 2016, Toledo-Hernández et al. 2016). This is also currently the case with Morimopsini, an assemblage of about 50 lamiine genera known mainly from the Afrotropical and Oriental Regions (Sudre and Teocchi 2002, Vitali and Menufandu 2010, Weigel 2015, Gouverneur 2016). Several taxa now assigned to Morimopsini occur also in Borneo (Breuning 1950, Gabriš et al. 2016). Pascoe (1886) described the genus *Anexodus* Pascoe for *A. aquilus* Pascoe, 1886 from Sabah. Additionally, Aurivillius (1911) described *Dolichostyrax* and *Pantilema* for *D. moultoni* Aurivillius, 1911 and *P. angustum* Aurivillius, 1911 from Sarawak and later, he added *D. longipes* Aurivillius, 1913 from Sabah (Aurivillius 1913). Kriesche (1924) described *Anexodus kuntzeni* Kriesche, 1924 based on three specimens from Mt. Kinabalu. Breuning (1950) made a key to the World Morimopsini and synonymized *A. kuntzeni* with *A. aquilus*. Since then, nobody has published on the morimopsine fauna of Borneo until Sudre (1997) described *Anexodus sarawakensis* Sudre, 1997 based on three specimens from Sarawak. Recently, Gabriš et al. (2016) revised the Bornean species of *Dolichostyrax* and described four species from Sabah for which they established three new genera, i.e. *Borneostyrax*, *Eurystyrax*, and *Microdolichostyrax*. They also reported ovoviviparity for the first time in Cerambycidae, when they found large larvae within the females of *Borneostyrax cristatus* Gabriš, Kundrata & Trnka, 2016.

To finish a revision of the genera classified in Morimopsini in Borneo, we herein review the species of *Anexodus* and *Pantilema*. For the first time, male and female genitalia are investigated and an identification key is provided for the species of *Anexodus*.

## Material and methods

In this study we examined mounted adults of both sexes. Genitalia were briefly submerged in hot 10% KOH, dissected and transferred to glycerol. Main diagnostics were photographed using a Zeiss Discovery.V12 with ZEN software. The line illustrations were derived from the photographs. All dissected parts were mounted on separate cardboards using Dimethyl Hydantoin Formaldehyde (DMHF) resin and pinned under the specimens. The measurements of taxonomically relevant morphological structures were taken with a measuring tool in ZEN software as follows: body length (BL) measured from the fore margin of head to the apex of elytra; body width (BW), pronotal width at the widest part; pronotal length at midline. Data from the locality labels are cited verbatim. A slash (/) is used to separate lines on the same label and a double slash (//) is used to separate different labels on the pin. The morphological terminology is used as in Gabriš et al. (2016), following those in Ślipiński and Escalona (2013) and Švácha and Lawrence (2014).

### Depositories

**BMNH** Natural History Museum, London, The United Kingdom (M. Barclay, M. Geiser)

**MHNG** Muséum d’Histoire Naturelle, Geneva, Switzerland (G. Cuccodoro)

**MNHUB** Museum für Naturkunde, Humboldt-Universtät Berlin (J. Willers)

**NHRS** Swedish Museum of Natural History, Stockholm, Sweden (J. Bergsten)

**PCDH** personal collection of Daniel J. Heffern, Houston, TX, USA

**PCJC** personal collection of James S. Cope, San Jose, CA, USA

**UBDC** Universiti Brunei Darussalam, Brunei

**UPOL** Palacky University, Olomouc, Czech Republic

## Taxonomy

### Genus Anexodus

Taxon classificationAnimaliaColeopteraCerambycidae

Pascoe, 1886

Anexodus Pascoe, 1886: 242.

#### Type species.

*Anexodus aquilus* Pascoe, 1886.

#### Differential diagnosis.

This genus is easily recognizable among the Bornean Morimopsini by its antennae, which are always shorter than body (Figs 1–8), and with antennomere II distinctly longer than antennomere III (Figs 21–27).

#### Description.

Body elongate to elongate-oval, small to medium-sized. Body densely clothed with very short pubescence; coloration either more or less uniformly brown or brown with yellowish stripes ranging from vertex through sides of pronotum to basal part of elytra, mouthparts lighter; in some cases antennae reddish brown or black (Figs 1–16).

Head about the same width as anterior margin of pronotum; genae sub-parallel at frontal view; frontoclypeus with distinct midline running from interantennal groove to labrum, sparsely covered with large, rounded, deep punctures; antennal tubercles prominent with deep narrow depression in between; antennal cavities opened dorsally; anterior margin of anteclypeus shallowly emarginate, with sparse long yellowish semi-erect setae. Labrum free, transverse, glabrous, either with one row of punctures bearing long setae (Figs 18–19) or with whole surface moderately sparsely, irregularly punctured (Figs 17, 20). Eyes small, reniform, vertically elongate, more or less emarginate at antennal articulations, lower parts distinctly narrower than genae. Antennae filiform, 11-segmented, shorter than body in both sexes; scape and pedicel covered with very short dense pubescence; the rest of antennomeres with much sparser pubescence; scape enlarged, swollen, slightly curved, longest, reaching at most center of pronotum, subparallel-sided, gradually slightly widened towards apex, thickest at apical part, apex either simple (Figs 23–27) or with distinct lateral hook-shaped projection (Figs 21–22), pedicel very long, apical antennomere simple, about two times as long as penultimate antennomere. Mandibles short and broad, apex unidentate (Fig. 17–20). Maxillary palpi tetramerous, apical palpomere fusiform (Figs 17–20). Labial palpi trimerous, apical palpomere of same shape as maxillary one.

Prothorax subcylindrical, about as long as wide, widest before middle, then gradually narrowed towards posterior margin, laterally with one small more or less distinct tubercle; pronotal disc weakly convex, sparsely covered with deep punctures, with indistinct tubercles, anterior and posterior angles obtuse. Prosternum in front of coxae slightly shorter than diameter of coxal cavity, procoxal cavities circular, with lateral extension, narrowly separated. Scutellum transverse, widely rounded apically, about two times as wide as long. Elytra elongate, 1.6–1.8 times as long as wide at widest part, 1.7–2.1 times as long as pronotum in males and 2.0–2.3 times in females, basally slightly wider than posterior pronotal margin, widest near middle, from middle gradually tapered towards apex, fused along suture; each elytron with three rows of tubercles irregular in size, in some cases inner row forming a distinct ridge basally (Figs 3, 11), sparsely covered by large deep punctures arranged irregularly in rows; outer elytral margin curved at lateral view (Figs 9–16). Mesoventrite with anterior edge on different plane than metaventrite; mesocoxal cavities circular. Metaventrite transverse, more than two times as wide as long, posterior margin with more or less narrow, deep median groove. Metacoxal cavities separated as widely as mesocoxal ones, extending laterally to meet elytra. Hind wings absent. Legs long, slender; femora weakly swollen distally, tibial spurs 2-2-2, protibiae with pubescent groove (antennal cleaner) on inner face, mesotibiae with pubescent groove on outer face, metatibiae without groove; tarsal formula 4-4-4; last tarsomere with four long erected setae at ventral face, claws simple, empodium absent.

Abdomen with five ventrites (Figs 9–16), first ventrite (excluding intercoxal process) almost two times longer than second; intercoxal process broadly rounded apically. Fifth ventrite with apex rounded in males and truncate in females, margin with sparse semi-erect pubescence. Male genitalia with tegmen elongate, widest near middle, basally with more or less short strut; parameres elongate, setose apically (Figs 28–31). Penis subparallel-sided, apically truncate or subacute; dorsal struts diverged from about 1/2 of penis length. Internal sac long, with paired small medial sclerites and distinct flagellar sclerites. Female genitalia with ovipositor elongate, narrow, apically with short styli. Vagina narrow, with pair of vaginal plates. Spermatheca present, more or less sclerotized, slender, elongate, curved; sclerotized part of spermathecal duct simple or strongly coiled (Figs 32–34).

### Anexodus aquilus

Taxon classificationAnimaliaColeopteraCerambycidae

Pascoe, 1886

Figs 1–3 , 9–11 , 17 , 21 , 22 , 28 , 32

Anexodus aquilus Pascoe, 1886: 242.
Anexodus kuntzeni Kriesche, 1924: 291; synonymized by Breuning (1950): 258.

#### Type material examined

(*A. aquilus*). Holotype, male, [Malaysia] “Type [circular label with red margin, printed] // N / Borneo [blue oval label, handwritten] // *Anexodus* / *aquilus* / type Pasc. [handwritten] // *Anexodus* / *aquilus* / N. Borneo Pa [handwritten] // Pascoe / Coll. / 93-60” (BMNH).

**Figures 1–4.**
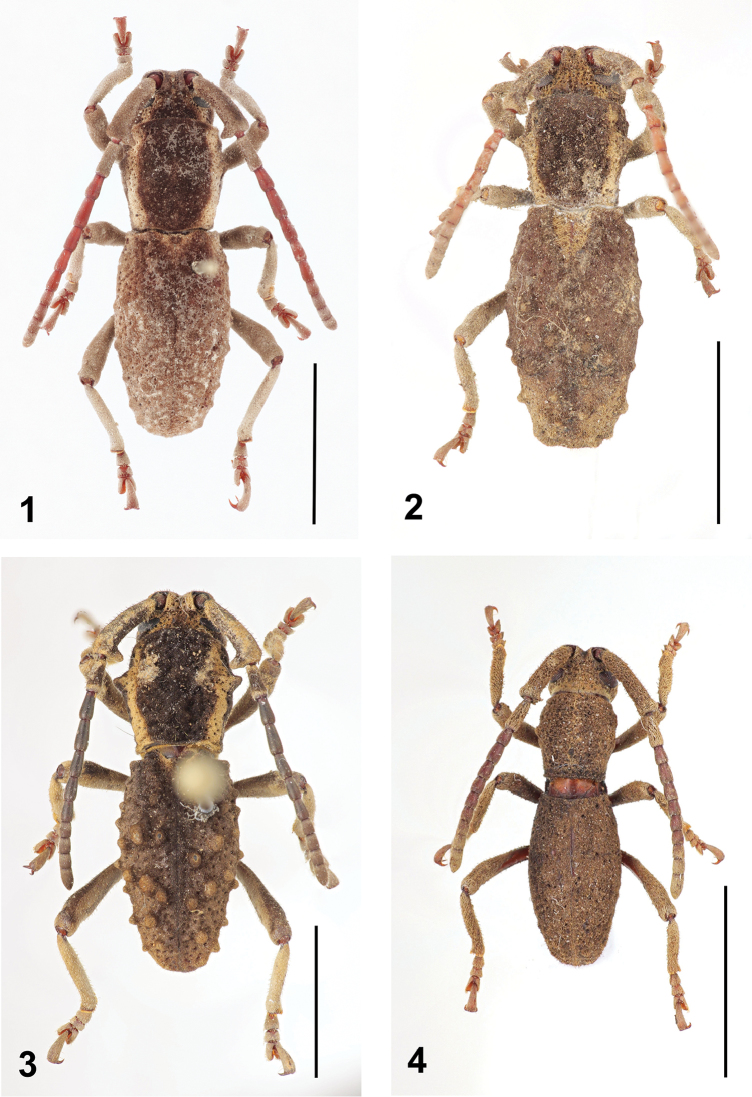
Dorsal habitus of *Anexodus* spp.: **1** *Anexodus aquilus* Pascoe, holotype male **2** *Anexodus aquilus* Pascoe, female (= holotype of *Anexodus kuntzeni* Kriesche) **3** *Anexodus aquilus* Pascoe, large male from Trus Madi **4** *Anexodus syptakovae* sp. n., holotype male. Scale bar = 5 mm.

#### Type material examined

(*A. kuntzeni*). Holotype, female, [Malaysia] “N:Borneo / Kina-Balu-Geb. / Waterstradt S. [printed] // *Anexodus* / *küntzeni* Kriesche / Typ! [handwritten] // *Anexodus* / *aquilus* Pasc. / Breuning dét. // HOLOTYPUS / *Anexodus* / *kuntzeni* Kriesche 1924 / labelled by MNHUB 2014 [red label, printed]” (MNHUB); male, “N:Borneo / Kina-Balu-Geb. / Waterstradt S. [printed] // *Anexodus* / *küntzeni* Kriesche / Paratyp! [handwritten] // PARATYPUS / *Anexodus* / *kuntzeni* Kriesche 1924 / labelled by MNHUB 2014 [red label, printed]” (MNHUB); female, “N. Borneo / Kinabalů [handwritten] // *Anexodus* / *küntzeni* [handwritten] // *Anexodus* / *aquilus* Pasc. / Breuning dét. // PARATYPUS / *Anexodus* / *kuntzeni* Kriesche 1924 / labelled by MNHUB 2014” (MNHUB).

#### Other material examined.

Female, “Malaysia, Sabah, / Crocker Range 20- / IV-2007 Cope / collection” (PCJC); female, “Malaysia, Sabah / Crocker Range / III-22-2009 / local coll” (PCDH); female, “Malaysia, Sabah / Sandakan / II-12-2007 / local coll // *Anexodus* / *aquilus* Pasc. / det. D. Heffern ‘07” (PCDH); female, “Malaysia, Sabah / Ranau 700 m / IV-23-2015 / local coll” (PCDH); female, “Malaysia, Sabah / Tenom / III-2-2008 / local coll” (PCDH); male, “Malaysia, Sabah, Mt. / Trus Madi 26-IV-2010 / Cope Collection” (PCJC); male, “Malaysia, Sabah, Mt. / Trus Madi 26-V-2012 / Cope Collection” (PCJC); male, “Malaysia, Sabah / Mt. Trus-Madi / IV-15-2005 / local coll // *Anexodus* / *aquilus* / Pascoe / det. J. Sudre” (PCDH).

#### Differential diagnosis.

This species is similar to *A. tufi* sp. n. in general habitus, yellowish stripes on dorsal body surface and a labrum with the entire surface punctured but differs in having apex of scape with a distinct lateral hook-shaped projection (simple in *A. tufi* sp. n.; Figs 21, 22, 26, 27), relatively longer parameres (Fig. 28), and a widened second half of spermatheca (Fig. 32).

#### Description.

Holotype (male). BL 11.5 mm, BW 3.8 mm. Body brown with yellowish stripes extending from vertex through sides of pronotum to basal part of elytra; mouthparts lighter; antennae reddish brown. Body densely clothed with very short brown pubescence. Head about as wide as anterior margin of pronotum. Labrum transverse, with whole surface moderately sparsely, irregularly punctured (Fig. 17). Eyes moderately emarginate at antennal articulations (Figs 9–11). Antennae 0.9 times as long as body; scape gradually slightly widened towards apex, thickest at apical part, apex with a distinct lateral hook-shaped projection (Fig. 21–22); the relative ratio of antennomere lengths 3.3 : 1.6 : 1.0 : 1.2 : 1.1 : 0.9 : 0.8 : 0.6 : 0.6 : 0.6 : 1.1.

Prothorax as long as wide, laterally with one distinct tubercle; pronotal disc with a pair of indistinct tubercles near middle and one median at second half; pronotal tubercles punctate. Prosternum in front of coxae 0.9 times shorter than diameter of coxal cavity. Scutellum transverse, two times as wide as long. Elytra elongate, 1.6 times as long as wide at widest part, 1.7 times as long as pronotum, widest near middle; each elytron with three rows of indistinct tubercles, inner row forming a distinct ridge basally; sparsely covered with large deep punctures arranged in slightly irregular rows. Legs long, slender; relative lengths of metatarsomeres 1.0 : 0.5 : 1.0 : 2.0.

Male genitalia with tegmen elongate, widest near middle, basally with very short strut; parameres elongate, 3.5 times longer than wide, apically with long setae (Fig. 28). Penis subparallel-sided, apically truncate; dorsal struts diverged from about 1/3 of penis length. Internal sac long, with paired small medial sclerites and distinct flagellar sclerites.

Variability in males. BL 9.1–12.5 mm, BW 3.1–4.2 mm. Antennae are either reddish brown, brown or black. There is a gradual morphological variation in the pronotal and elytral tubercles, ranging from the less distinct tubercles in the holotype (Figs 1, 9) through the more distinct tubercles in most specimens to the strongly developed tubercles with inner elytral row forming a conspicuous ridge basally in the specimens from Trus Madi (Figs 3, 11).

Female. Most characters same as for males. BL 10.0–13.0 mm, BW 3.2–4.5 mm. Body more convex dorsally, with distinct tubercles on pronotum and elytra. Antennae shorter, 0.6–0.7 times as long as body. Elytra 1.7 times as long as wide, 2.0–2.1 times as long as pronotum. Spermatheca sclerotized, slender, elongate, curved, widened at second half, gradually tapered toward apex; sclerotized part of spermathecal duct simple (Fig. 32).

#### Distribution.

Malaysia: Borneo (Sabah; Fig. 43).

### Anexodus sarawakensis

Taxon classificationAnimaliaColeopteraCerambycidae

Sudre, 1997

Figs 5–6 , 13–14 , 18 , 23–24 , 29 , 34

Anexodus sarawakensis Sudre, 1997: 253.

#### Type material examined.

Holotype, male, “E. MALAYSIA: Sarawak / confl. Sun Oyan and / Mujong riv., E. Kapit / 50m, 18.V.1994, # 5 / Löbl & Burckhardt // Holotype // *Anexodus* / *sarawakensis* Nov sp. / J. Sudre det. 1996” (MHNG). Paratype, female, “E. MALAYSIA: Sarawak / confl. Sun Oyan and / Mujong riv., E. Kapit / 50m, 18.V.1994, # 5 / Löbl & Burckhardt // comparé zu type / d’*A. aquilus* per / J. Sudre 1996 // Paratype // *Anexodus* (♀) / *sarawakensis* sp. n / J. Sudre det. 1996” (MHNG).

**Figures 5–8.**
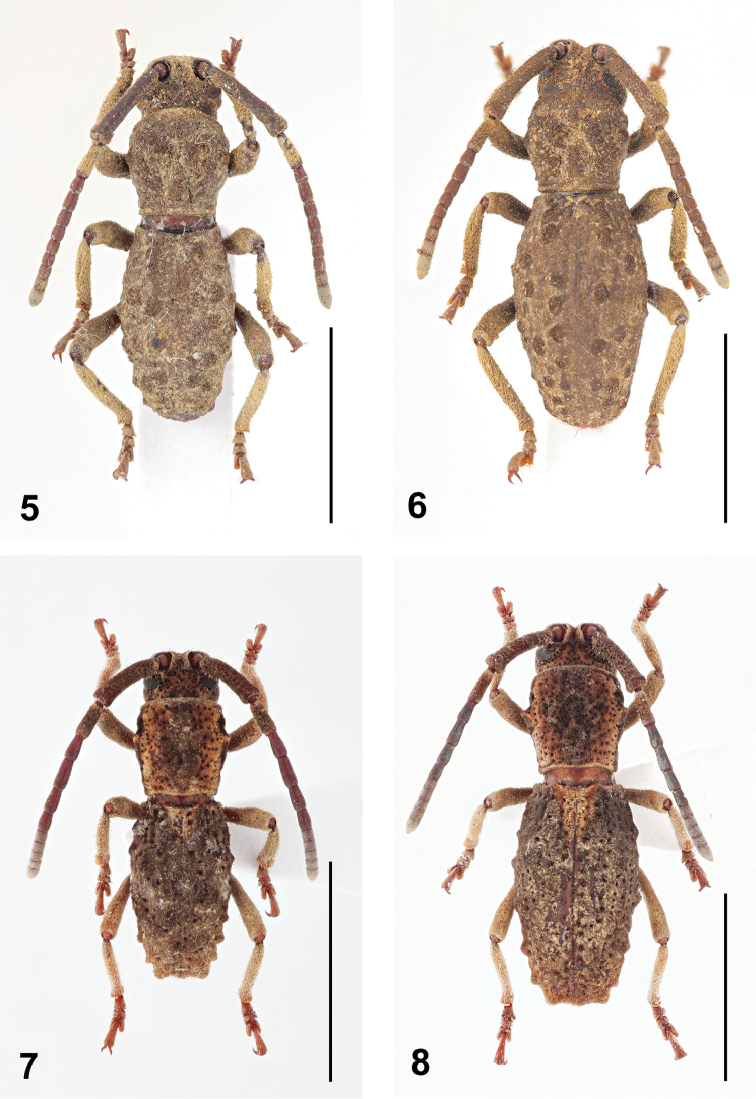
Dorsal habitus of *Anexodus* spp.: **5** *Anexodus sarawakensis* Sudre, holotype male **6** *Anexodus sarawakensis* Sudre, paratype female **7** *Anexodus tufi* sp. n., holotype male **8** *Anexodus tufi* sp. n., paratype female. Scale bar 5 mm.

#### Differential diagnosis.

*Anexodus sarawakensis* is similar to *A. syptakovae* sp. n. in having uniformly colored habitus and labrum with one row of distinct punctures with setae (Fig. 18). For more details see the differential diagnosis under the latter species.

#### Description.

Holotype (male). BL 9.2 mm, BW 3.1 mm. Body uniformly brown, mouthparts lighter. Body densely clothed with very short brown pubescence. Head about as wide as anterior margin of pronotum. Labrum transverse, with one row of punctures bearing long setae (Fig. 18). Eyes less emarginate at antennal insertions (Figs 13–14). Antennae 0.8 times as long as body; scape gradually slightly widened towards apex, thickest at apical part, apex simple (Figs 23–24); the relative ratio of antennomere lengths 6.3 : 2.5 : 1.0 : 1.3 : 1.1 : 1.0 : 1.0 : 0.9 : 0.8 : 1.0 : 2.0.

Prothorax as long as wide, laterally with one moderately distinct tubercle; pronotal disc with a pair of distinct tubercles near middle and one median at second half; pronotal tubercles punctate. Prosternum in front of coxae 0.9 times shorter than diameter of coxal cavity. Scutellum transverse, two times as wide as long. Elytra elongate, 1.6 times as long as wide at widest part, 1.8 times as long as pronotum, widest near middle; each elytron with three rows of distinct tubercles (Figs 5–6), sparsely covered with large deep punctures arranged in slightly irregular rows. Legs long, slender; relative lengths of metatarsomeres 1.0 : 0.7 : 1.1 : 2.0.

**Figures 9–16.**
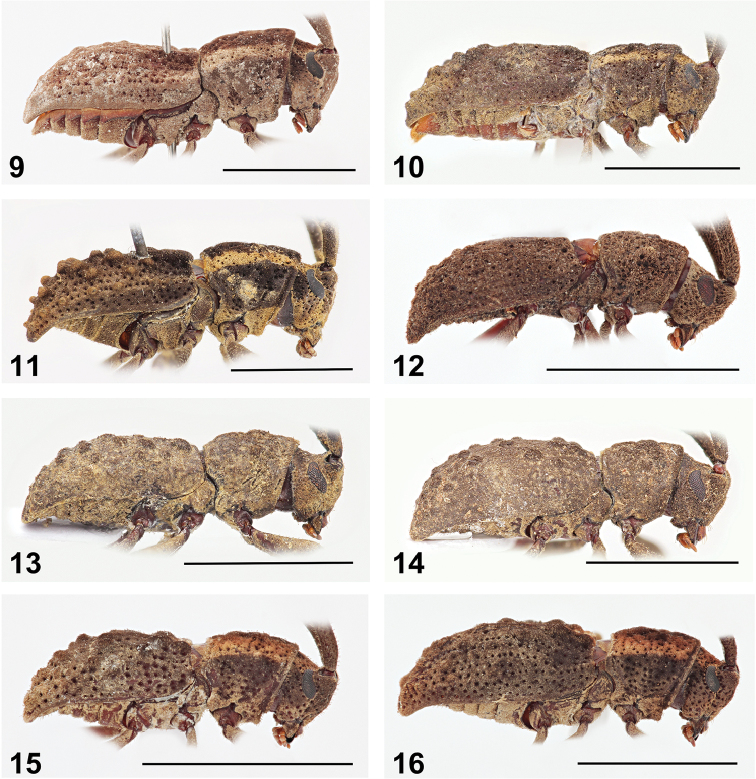
Lateral habitus of *Anexodus* spp.: **9** *Anexodus aquilus* Pascoe, holotype male **10** *Anexodus aquilus* Pascoe, female (= holotype of *Anexodus kuntzeni* Kriesche) **11** *Anexodus aquilus* Pascoe, large male from Trus Madi **12** *Anexodus syptakovae* sp. n., holotype male **13** *Anexodus sarawakensis* Sudre, holotype male **14** *Anexodus sarawakensis* Sudre, paratype female **15** *Anexodus tufi* sp. n., holotype male **16** *Anexodus tufi* sp. n., paratype female. Scale bar 5 mm.

Male genitalia with tegmen elongate, widest near middle, basally with short strut; parameres elongate, 2.9 times longer than wide, apically with long setae (Fig. 29). Penis subparallel-sided, apically subacute; dorsal struts diverged from about one third of penis length. Internal sac long, with paired small medial sclerites and distinct flagellar sclerites.

Female. Most characters same as for males. BL 9.9 mm, BW 3.5 mm. Body more convex dorsally. Antennae slightly shorter than in male, 0.7 times as long as body; length ratio of antennomeres I–III: 6.5 : 2.7 : 1.0. Elytra 1.7 times as long as wide, 2.3 times as long as pronotum. Spermatheca only slightly sclerotized, slender, elongate; sclerotized part of spermathecal duct strongly coiled (Fig. 34).

#### Distribution.

Malaysia: Borneo (Sarawak: Kapit; Fig. 43).

### Anexodus syptakovae sp. n.

Taxon classificationAnimaliaColeopteraCerambycidae

http://zoobank.org/DC100C9A-EB88-43D9-A812-CA8C4A20FEEE

Figs 4 , 12 , 19 , 25 , 30

#### Type material.

Holotype, male, [Malaysia] “SARAWAK: / 5th Division / Gn. Mulu NP // Camp 5 / Kerangas // Pitfall / trap // iv. 78, N. M. Collins / B.M.1978-11 // *Opsies* sp.” (BMNH). Paratype, male, “SARAWAK: / 4th Division / Gn. Mulu NP // mixed / dipterocarp / forest // Site B / 130 m. // soil cores // N. M. Collins / B.M.1978-11” (BMNH).

#### Differential diagnosis.

This species is similar to *A. sarawakensis* in having uniformly colored habitus and labrum with a row of distinct punctures with setae (Fig. 19). However, *A. syptakovae* sp. n. is smaller, with different ratio of antennomeres I–III (Figs 23–25), less distinct tubercles on pronotal disc and elytra (Fig. 4), and relatively shorter and wider parameres, with tufts of shorter setae apically (longer parameres with longer setae apically in *A. sarawakensis*; Figs 29–30).

#### Description.

Holotype (male). BL 8.0 mm, BW 2.5 mm. Body uniformly brown, mouthparts lighter. Body densely clothed with very short brown pubescence. Head slightly wider than anterior margin of pronotum. Labrum transverse, with one row of punctures bearing long setae (Fig. 19). Eyes less emarginate at antennal insertions (Fig. 12). Antennae 0.9 times as long as body; scape gradually slightly widened towards apex, thickest at apical part, apex simple (Fig. 25); the relative ratio of antennomere lengths 4.4 : 1.7 : 1.0 : 1.1 : 1.0 : 0.9 : 0.9 : 0.8 : 0.7 : 0.9 : 1.4.

Prothorax as long as wide, laterally with one indistinct tubercle; pronotal disc with a pair of very indistinct tubercles near middle and one median at second half; pronotal tubercles punctate. Prosternum in front of coxae 0.9 times shorter than diameter of coxal cavity. Scutellum transverse, about two times as wide as long. Elytra elongate, 1.8 times as long as wide at widest part, 2.1 times as long as pronotum, widest near middle; each elytron with three rows of only slightly elevated tubercles (Fig. 12), sparsely covered with large deep punctures arranged in slightly irregular rows. Legs long, slender; relative lengths of metatarsomeres 1.0 : 0.7 : 1.1 : 1.9.

Male genitalia with tegmen elongate, widest near middle, basally with short strut; parameres elongate, 2.3 times longer than wide, apically with tufts of short setae (Fig. 30). Penis subparallel-sided, apically truncate; dorsal struts diverged from about half of penis length. Internal sac long, with paired small medial sclerites and distinct flagellar sclerites.

#### Intraspecific variability.

Paratype is smaller (BL 7.00 mm, BW 2.3 mm), with reddish brown antennae.

Female unknown.

#### Distribution.

Malaysia: Borneo (Sarawak: Gn. Mulu NP; Fig. 43).

#### Etymology.

The specific name is a matronym in honor of Ms. Hana Gabriš Syptáková (Salisov, Czech Republic).

### Anexodus tufi sp. n.

Taxon classificationAnimaliaColeopteraCerambycidae

http://zoobank.org/85AFEBB7-502B-4303-8C86-B9DD45AD8F1F

Figs 7–8 , 15–16 , 20 , 26–27 , 31 , 33 , 40

#### Type material.

Holotype, male, “BRUNEI, Ulu Temburong NP / Kuala Belalong FSC / 4°32'47.6"N 115°09'27"E / I. H. Tuf leg. II.2013” (UBDC); paratype, female, same data as holotype (UPOL); 2 paratypes, females, “BRUNEI, Ulu Temburong NP / Kuala Belalong FSC / 4°32'47.6"N 115°09'27"E / Z. Mačát leg. I.2014” (BMNH); paratype, female, “BRUNEI, Ulu Temburong NP / Kuala Belalong FSC / 4°32'47.6"N 115°09'27"E / O. Machač leg. II.2015” (UPOL).

#### Differential dagnosis.

This species is similar to *A. aquilus* in having yellowish stripes on the dorsal body surface and labrum with the whole surface punctured (Fig. 20), but it differs in shape of the scape (apex simple in *A. tufi* sp. n., apex with distinct lateral hook-shaped projection in *A. aquilus*; Figs 21–22, 26–27), length of the parameres (relatively longer in *A. aquilus*; Figs 28, 31), and shape of the spermatheca (simply elongated in *A. tufi* sp. n., widened at second half in *A. aquilus*; Figs 32–33).

**Figures 17–27.**
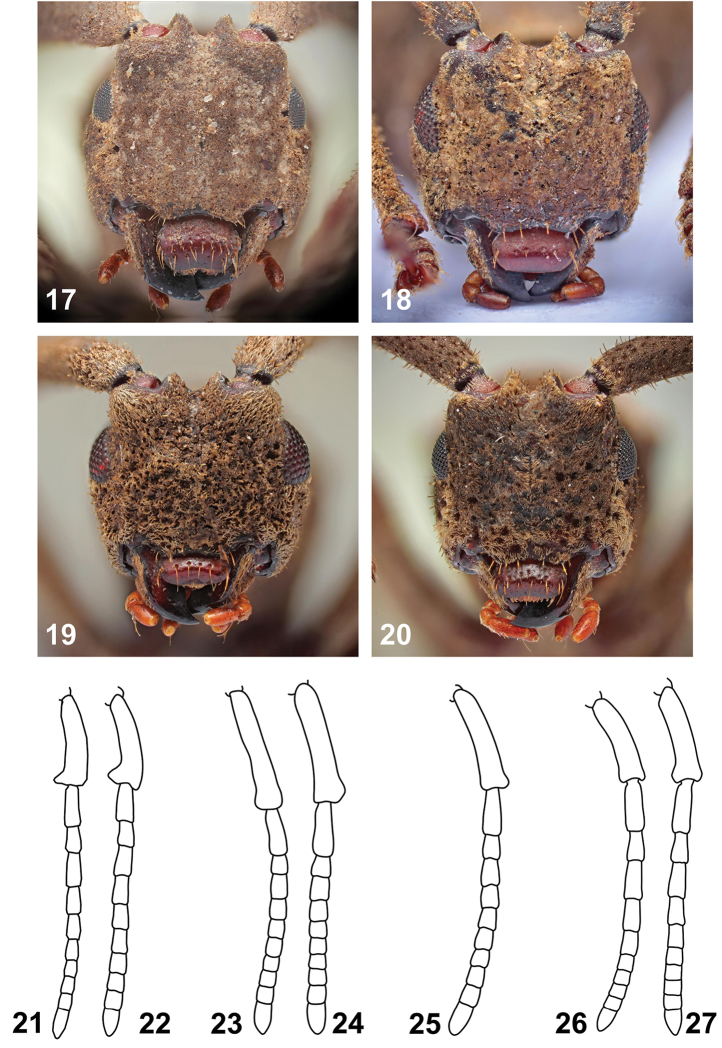
*Anexodus* spp.: **17–20** Head, frontal view: **17** *Anexodus aquilus* Pascoe, holotype male **18** *Anexodus sarawakensis* Sudre, holotype male **19** *Anexodus syptakovae* sp. n., holotype male **20** *Anexodus tufi* sp. n., holotype male **21–27** Antenna: **21** *Anexodus aquilus* Pascoe, male **22** *Anexodus aquilus* Pascoe, female **23** *Anexodus sarawakensis* Sudre, male **24** *Anexodus sarawakensis* Sudre, female **25** *Anexodus syptakovae* sp. n., male **26** *Anexodus tufi* sp. n., male **27** *Anexodus tufi* sp. n., female. Not to scale.

#### Description.

Holotype (male). BL 7.4 mm, BW 2.3 mm. Body brown with yellowish stripes ranging from vertex through sides of pronotum to basal part of elytra, densely clothed with very short brown pubescence. Head about as wide as anterior margin of pronotum. Labrum transverse, its surface with moderately sparse punctures (Fig. 20). Eyes moderately emarginate at antennal articulations (Figs 15–16). Antennae 0.9 times as long as body; scape gradually slightly widened towards apex, thickest at apical part, apex simple (Figs 26–27); the relative ratio of antennomere lengths 3.2 : 1.5 : 1.0 : 1.2 : 1.0 : 0.9 : 0.5 : 0.5 : 0.5 : 0.5 : 1.0.

Prothorax as long as wide, laterally with one distinct tubercle; pronotal disc with a pair of moderately distinct tubercles near middle and one median at second half and one indistinct median at anterior half; pronotal tubercles punctate. Prosternum in front of coxae 0.9 times shorter than diameter of coxal cavity. Scutellum transverse, two times as wide as long. Elytra elongate, 1.8 times as long as wide at widest part, 1.9 times as long as pronotum, widest near middle; each elytron with three rows of distinct, longitudinally elongate tubercles (Figs 7–8, 15–16), inner row forming a distinct ridge basally; sparsely covered with large deep punctures arranged in slightly irregular rows. Legs long, slender; relative lengths of metatarsomeres 1.0 : 0.6 : 1.0 : 2.1.

Male genitalia with tegmen elongate, widest before middle, basally with short strut; parameres elongate, 3.3 times longer than wide, apically with long setae (Fig. 31). Penis subparallel-sided, apically truncate; dorsal struts diverged from about 1/3 of penis length. Internal sac long, with paired small medial sclerites and distinct flagellar sclerites.

#### Intraspecific variability.

The male paratype is larger (body length 9.4 mm, body width 2.8 mm).

Female. Most characters same as for males. BL 8.5–10.2 mm, BW 2.7–3.4. Antennae shorter, 0.7 times as long as body, with relatively longer scape and pedicel (length ratio of antennomeres I–III: 3.4–3.6 : 1.7–1.8 : 1.0). Elytra 2.2–2.3 times as long pronotum. Fifth ventrite with apex truncate. Spermatheca sclerotized, slender, elongate, curved, gradually tapered toward apex; sclerotized part of spermathecal duct simple (Fig. 33).

**Figures 28–34.**
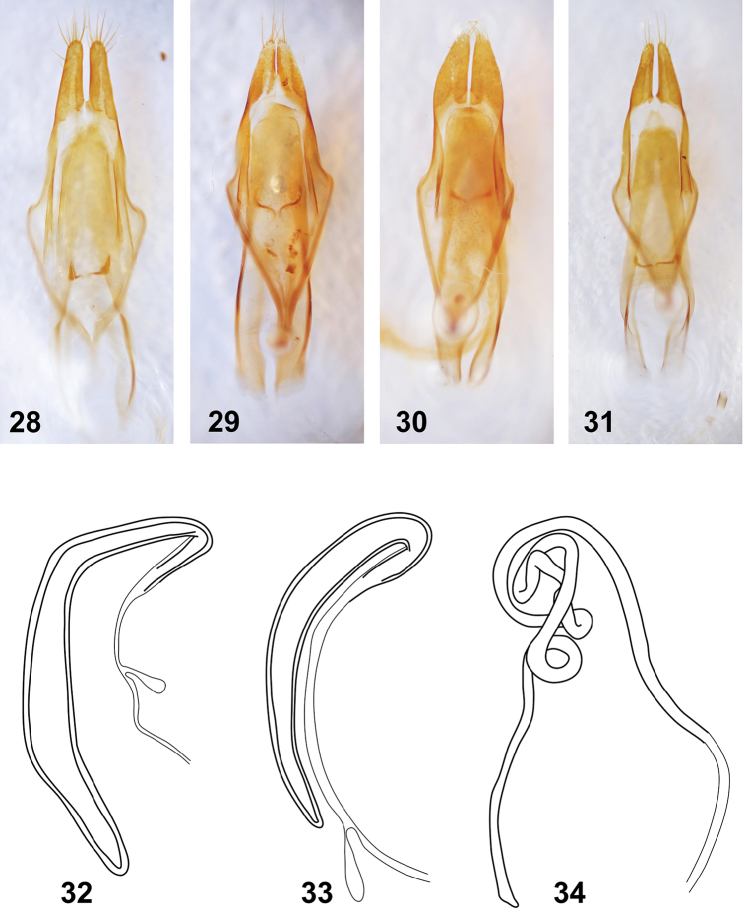
*Anexodus* species: **28–31** Aedeagus, ventral view: **28** *Anexodus aquilus* Pascoe, holotype **29** *Anexodus sarawakensis* Sudre, holotype **30** *Anexodus syptakovae* sp. n., holotype **31** *Anexodus tufi* sp. n., holotype **32–34** Spermatheca: **32** *Anexodus aquilus* Pascoe **33** *Anexodus tufi* sp. n. **34** *Anexodus sarawakensis* Sudre. Not to scale.

#### Distribution.

Brunei (Fig. 43). All the specimens in the type series are from the vicinity of the Kuala Belalong Field Studies Centre (KBFSC) in the Ulu Temburong National Park (Fig. 41) which has been described in detail by Ševčík et al. (2014).

#### Etymology.

This species is named after Mr. I. H. Tuf (UPOL, Czech Republic; Fig. 42), who collected a part of the type series.

### Genus Pantilema

Taxon classificationAnimaliaColeopteraCerambycidae

Aurivillius, 1911

Pantilema Aurivillius, 1911: 196.

#### Type species.

*Pantilema angustum* Aurivillius, 1911.

#### Differential diagnosis.

**Figures 35–39.**
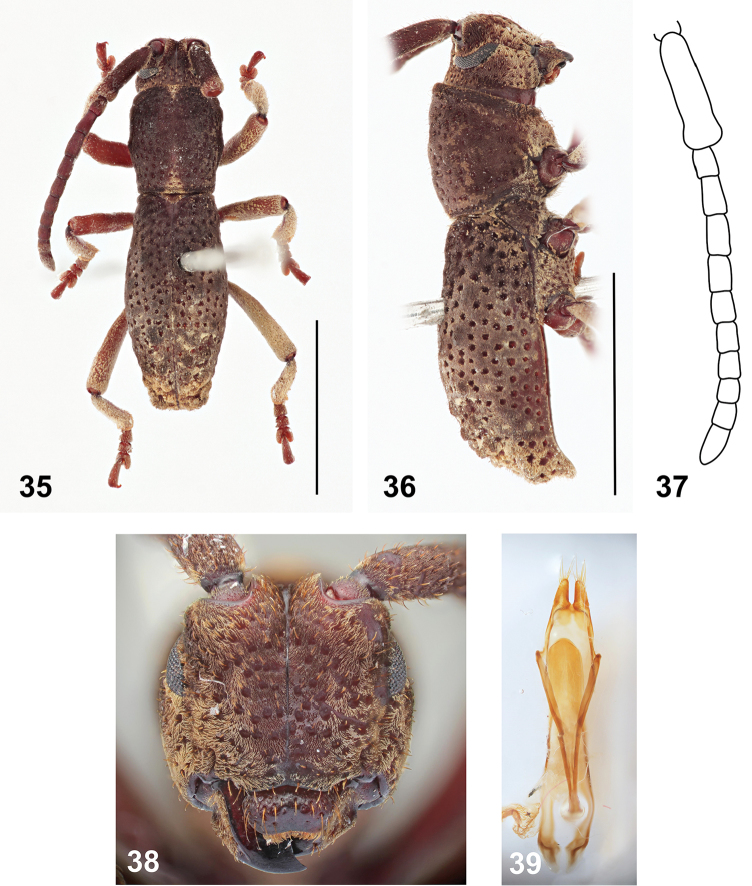
*Pantilema angustum* Aurivillius, holotype male: **35** Dorsal habitus (scale bar 5 mm) **36** Lateral habitus (scale bar 5 mm) **37** Antenna **38** Head, frontal view **39** Aedeagus, ventral view. Scale bar 5 mm.

*Pantilema* differs from the remaining Bornean Morimopsini by having a slender, narrow, parallel-sided body (body length/width ratio = 3.5), tibial spurs 1-1-2, truncate elytral apex, and tubercles only in the apical half of the elytra (Figs 35–36).

#### Description.

Body slender, elongate, densely clothed with very short pubescence; coloration brown, with some parts paler, yellowish, antennae and legs reddish-brown (Figs 35–36).

Head about the same width as anterior margin of pronotum; genae convex at frontal view; frontoclypeus with distinct midline running from interantennal groove to labrum, sparsely punctured; antennal tubercles prominent with narrow, deep depression in between; antennal cavities opened dorsally; anterior margin of anteclypeus shallowly emarginate, with sparse long yellowish semi-erect setae. Labrum free, transverse, glabrous, with a row of distinct punctures and sparsely and irregularly distributed additional less distinct punctures, with sparse long semi-erect setae (Fig. 38). Eyes small, distinctly elongate vertically, narrow, about four times as long as wide, slightly emarginate at antennal insertions, lower parts distinctly narrower than genae (Fig. 36). Antennae filiform, 11-segmented, shorter than body; scape and first half of pedicel covered with very short dense light brown pubescence; the rest of antenna with much sparser pubescence; scape enlarged, swollen, reaching the first half of pronotum, subparallel-sided, apically slightly widened, pedicel short, apical antennomere simple, less than two times as long as penultimate antennomere (Fig. 37). Mandibles short and broad, apex unidentate (Fig. 38). Maxillary palpi tetramerous, apical palpomere fusiform. Labial palpi trimerous, apical palpomere of same shape as maxillary one.

Prothorax about as long as wide, subparallel-sided at anterior half, widest slightly medially, then gradually narrowed towards posterior margin, laterally with one very weakly developed tubercle; pronotal disc weakly convex, sparsely covered with deep punctures, not smooth, without tubercles (Fig. 38), anterior and posterior angles obtuse. Prosternum in front of coxae slightly shorter than diameter of coxal cavity, procoxal cavities circular, with lateral extension, narrowly separated. Scutellum transverse, subtriangular, about three times as wide as long. Elytra elongate, twice as long as wide at widest part, basally as wide as posterior pronotal margin, without distinct humeral bulges, widest near middle, fused along the elytral suture, apically truncate; with tubercles present only at apical third of elytra; tubercles arranged in two rows, apical tubercles forming large transverse irregularly shaped bulge (Fig. 35), elytra sparsely covered with large deep punctures arranged in slightly irregular rows; outer elytral margin distinctly curved at lateral view (Fig. 36). Mesoventrite with anterior edge on different plane than metaventrite; mesocoxal cavities circular, separated slightly wider than in procoxal cavities. Metaventrite transverse, more than two times as wide as long. Metacoxal cavities extending laterally to meet elytra. Hind wings absent. Legs long, slender; femora weakly swollen distally, tibial spurs 1-1-2, protibiae with pubescent groove (antennal cleaner) on inner face, mesotibiae with pubescent groove on outer face, metatibiae without groove; tarsal formula 4-4-4, last tarsomere with four long erected setae at ventral face, claws simple, empodium absent.

Abdomen with five visible ventrites, first ventrite (excluding intercoxal process) almost 1.5 times longer than second; intercoxal process subparallel-sided basally, narrowed and broadly rounded apically. Fifth ventrite with apex truncate, margin with sparse semi-erect pubescence. Male genitalia with tegmen elongate, widest at apical third, basally with long strut; parameres moderately long, setose apically. Penis relatively long, with dorsal struts diverged from about two fifths of penis length (Fig. 39). Internal sac long, with paired medial sclerites and distinct complex of flagellar sclerites.

### Pantilema angustum

Taxon classificationAnimaliaColeopteraCerambycidae

Aurivillius, 1911

Figs 35–39

Pantilema angustum Aurivillius, 1911: 196.

#### Type material examined.

Holotype, male, “Samarahan / June 1906 [handwritten] // Type. // NHRS-JLKB / 000022859 // 5184 / E94 +” (NHRS).

#### Redescription.

Holotype (male). BL 10.6 mm, BW 3.1 mm. Body brown, with antennae and legs reddish-brown, elytral apex and two median spots near anterior and posterior pronotal margins yellowish, mouthparts lighter (Figs 35–36). Body densely clothed with very short brown pubescence. Head about as wide as anterior margin of pronotum. Labrum with a row of distinct punctures and sparsely and irregularly distributed additional less distinct punctures, with sparse long semi-erect setae (Fig. 38). Eyes slightly emarginate at antennal insertions (Fig. 36); minimum interocular distance 1.9 times maximum eye diameter. Antennae (left present in whole length; right with antennomeres II–XI missing) 0.7 times as long as body; scape reaching the first half of pronotum, subparallel-sided, apically slightly widened, the relative ratio of antennomere lengths 2.9 : 0.7 : 1.0 : 1.1 : 0.9 : 0.8 : 0.7 : 0.7 : 0.6 : 0.7 : 1.3 (Fig. 37).

Prothorax 1.1 times as long as wide, laterally with one very weakly developed tubercle; pronotal disc without tubercles (Fig. 38). Prosternum in front of coxae 0.9 times as wide as diameter of coxal cavity. Scutellum transverse, about three times as wide as long. Elytra 2.0 times as long as wide at widest part, 1.9 times as long as pronotum, without distinct humeral bulges, apically truncate; with tubercles present only at apical third of elytra; tubercles arranged in two rows, apical tubercles forming large transverse irregularly shaped bulge (Fig. 35), elytra sparsely covered with large deep punctures arranged in slightly irregular rows (Fig. 36). Legs long, slender, relative lengths of metatarsomeres 1.0 : 0.6 : 1.0 : 1.8.

Male genitalia with tegmen elongate, widest at apical third, basally with long strut; parameres moderately long. Penis long, apically broadly rounded; dorsal struts diverged from about two fifths of penis length (Fig. 39). Internal sac long, with paired small medial sclerites and distinct complex of flagellar sclerites formed by plates of sclerotized spines.

#### Distribution.

Malaysia: Borneo (Sarawak: Samarahan; Fig. 43).

### Identification key for the species of *Anexodus* Pascoe

**Table:** 

1	Body uniformly brown (Figs 4–6); labrum with one row of distinct punctures with setae (Figs 18–19)	**2**
–	Body brown with yellowish stripes dorsally (Figs 1–3, 7–8); labrum with whole surface with sparse punctures (Figs 17, 20)	**3**
2	Body length 7.0–8.0 mm; length ratio of antennomeres I–III 4.4–4.6 : 1.7–1.9 : 1.0 (Fig. 25); less distinct tubercles on pronotal disc and elytra (Figs 4, 12); parameres wider, 2.3 times longer than wide, with tufts of short setae apically (Fig. 30); Malaysia: Sarawak (Fig. 43)	***A. syptakovae* sp. n.**
–	Body length 9.2–9.9 mm; length ratio of antennomeres I–III 6.3–6.5 : 2.5–2.7 : 1.0 (Figs 23–24); more distinct tubercles on pronotal disc and elytra (Figs 5–6, 13–14); parameres more elongated, 2.9 times longer than wide, with long setae apically (Fig. 29); Malaysia: Sarawak (Fig. 43)	***A. sarawakensis* Sudre**
3	Apex of the scape with distinct lateral hook-shaped projection (Figs 21–22); parameres relatively longer, 3.5 times longer than wide (Fig. 28); spermatheca widened at second half (Fig. 32); Malaysia: Sabah (Fig. 43)	***A. aquilus* Pascoe**
–	Apex of the scape simple (Figs 26–27); parameres relatively shorter, 3.3 times longer than wide (Fig. 31); spermatheca simple, elongated (Fig. 33); Brunei (Fig. 43)	***A. tufi* sp. n.**

### Checklist of the Morimopsini in Borneo

Genus *Anexodus* Pascoe, 1886

*A. aquilus* Pascoe, 1886 (Malaysia: Sabah; Fig. 43) (type species, by monotypy)

*A. sarawakensis* Sudre, 1997 (Malaysia: Sarawak; Fig. 43)

*A. syptakovae* sp. n. (Malaysia: Sarawak; Fig. 43)

*A. tufi* sp. n. (Brunei; Fig. 43)

Genus *Borneostyrax* Gabriš, Kundrata & Trnka, 2016

*B. cristatus* Gabriš, Kundrata & Trnka, 2016 (Malaysia: Sabah; Fig. 43) (type species, by original designation)

Genus *Dolichostyrax* Aurivillius, 1911

*D. longipes* Aurivillius, 1913 (Malaysia: Sabah; Fig. 43)

*D. moultoni* Aurivillius, 1911 (Malaysia: Sarawak; Fig. 43) (type species, by monotypy)

Genus *Eurystyrax* Gabriš, Kundrata & Trnka, 2016

*E. nemethi* Gabriš, Kundrata & Trnka, 2016 (Malaysia: Sabah; Fig. 43) (type species, by original designation)

Genus *Microdolichostyrax* Gabriš, Kundrata & Trnka, 2016

*M. hefferni* Gabriš, Kundrata & Trnka, 2016 (Malaysia: Sabah; Fig. 43) (type species, by original designation)

*M. minutus* Gabriš, Kundrata & Trnka, 2016 (Malaysia: Sabah; Fig. 43)

Genus *Pantilema* Aurivillius, 1911

*P. angustum* Aurivillius, 1911 (Malaysia: Sarawak; Fig. 43) (type species, by monotypy)

## Discussion

**Figures 40–42.**
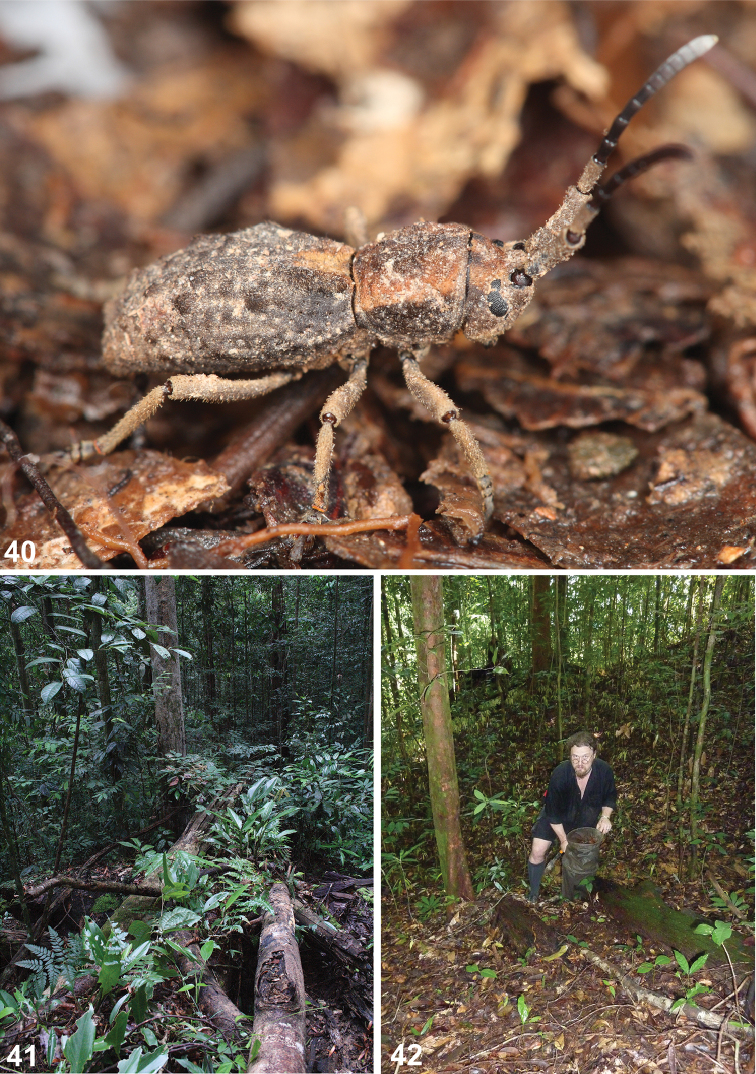
*Anexodus tufi* sp. n. in the Ulu Temburong National Park, Brunei: **40** Live specimen **41** Habitat near the Kuala Belalong Field Studies Centre **42** Ivan H. Tuf collecting invertebrates from the rainforest litter.

**Figure 43.**
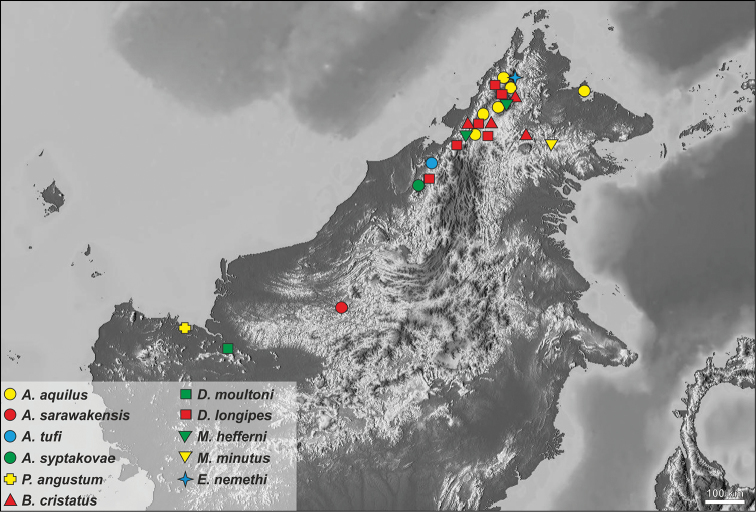
The distribution of Morimopsini in Borneo. A *Anexodus* Pascoe, B *Borneostyrax* Gabriš, Kundrata & Trnka, D *Dolichostyrax* Aurivillius, E *Eurystyrax* Gabriš, Kundrata & Trnka, M *Microdolichostyrax* Gabriš, Kundrata & Trnka, P *Pantilema* Aurivillius.

In 2013, two Czech universities (Palacky University in Olomouc, University of Ostrava) and the Universiti Brunei Darussalam established a collaboration which resulted in the biodiversity survey of the Ulu Temburong National Park in Brunei (Dančák et al. 2013; Ševčík et al. 2014; Hroneš et al. 2015; Ježek et al. 2015; Kočárek et al. 2015; Hippa et al. 2016; Kuřavová et al. 2017a, b). The collection of several specimens of *Anexodus* during the sifting of forest litter (Figs 40–42) encouraged a taxonomical revision of this genus and its relatives in Borneo. In the first part (Gabriš et al. 2016), the genus *Dolichostyrax* was revised, including the material identified by various researchers as belonging to that genus, and here, the remaining genera *Anexodus* and *Pantilema* are revised. Altogether, the occurrence of eleven species in six genera currently classified in Morimopsini in Borneo is confirmed. All known species are distributed in the northern part of Borneo (Fig. 43), which is the presumed Pleistocene rainforest refugium with a very high biodiversity (e.g. Gathorne-Hardy et al. 2002). As demonstrated by Gabriš et al. (2016) and here, the diversity of the morimopsine genera in Borneo is much higher than ever expected. These beetles are also often overlooked in the field due to their cryptic life-style (Figs 40–41) and because entomologists interested in Cerambycidae only rarely use sifting (Fig. 42) or pitfall traps as the collecting methods in the tropical forests. However, sifting forest leaf litter is an effective method for collecting various flightless beetle groups (e.g. Anderson and Ashe 2000; Kodada et al. 2013; Grebennikov 2014, 2016; Gerstmeier 2015) and its use in the Bornean rainforest could result in discoveries of further morimopsine lineages.

## Supplementary Material

XML Treatment for Anexodus

XML Treatment for Anexodus aquilus

XML Treatment for Anexodus sarawakensis

XML Treatment for Anexodus syptakovae

XML Treatment for Anexodus tufi

XML Treatment for Pantilema

XML Treatment for Pantilema angustum
